# IL-10 Signaling Elicited by Nivolumab-Induced Activation of the MAP Kinase Pathway Does Not Fully Contribute to Nivolumab-Modulated Heterogeneous T Cell Responses

**DOI:** 10.3390/ijms222111848

**Published:** 2021-10-31

**Authors:** Taylor A. Harper, Silvia M. Bacot, Christie Jane Fennell, Rebecca L. Matthews, Christina Zhu, Peng Yue, Alexander Benton, Devira Friedman, Adovi Akue, Mark A. KuKuruga, Shiowjen Lee, Tao Wang, Gerald M. Feldman

**Affiliations:** 1 Office of Biotechnology Products, Center for Drug Evaluation and Research, Food and Drug Administration, Silver Spring, MD 20993, USA; tah9h@virginia.edu (T.A.H.); Silvia.Bacot@fda.hhs.gov (S.M.B.); ChristieJane.Fennell@fda.hhs.gov (C.J.F.); rebecca.matthews@nih.gov (R.L.M.); Christina.Zhu@fda.hhs.gov (C.Z.); shandongyuepeng@hotmail.com (P.Y.); alexander.benton@okstate.edu (A.B.); dsf2141@barnard.edu (D.F.); 2 Office of Vaccines Research & Review, Center for Biologics Evaluation and Research, U.S. Food and Drug Administration, Silver Spring, MD 20993, USA; Adovi.Akue@fda.hhs.gov (A.A.); Mark.Kukuruga@fda.hhs.gov (M.A.K.); Shiowjen.Lee@fda.hhs.gov (S.L.)

**Keywords:** nivolumab, interleukin 10, T cells, cytokines, STAT3 pathway, AKT serine–threonine kinase pathway, MAP kinase pathway

## Abstract

Immune checkpoint inhibitor (ICI) therapy has revolutionized anti-cancer treatment for many late-stage cancer patients. However, ICI therapy has thus far demonstrated limited efficacy for most patients, and it remains unclear why this is so. Interleukin 10 (IL-10) is a cytokine that has been recognized as a central player in cancer biology with its ability to inhibit anti-tumor T cell responses. Recent studies suggest that IL-10 might also exert some intrinsic anti-tumor T cell responses, and clinical studies using recombinant IL-10 alone or in combination with ICI are underway. This paradoxical effect of IL-10 and its underlying mechanisms impacting ICI-modulated T cell responses remain poorly understood. In this study, using an in vitro mixed lymphocyte reaction assay, we found that treatment with ICIs such as the anti-programmed cell death receptor-1 (PD-1) mAb nivolumab elicits a strong expression of IL-10. While neutralization of IL-10 signaling with an anti-IL-10 specific mAb significantly decreases the production of IFN-γ by T cells in a cohort of donor cells, the opposite effect was observed in other donor cells. Similarly, neutralization of IL-10 signaling significantly decreases the expression of T cell activation markers Ki67 and CD25, as well as the production of Granzyme B in a cohort of donor cells, whereas the opposite effect was observed in others. Furthermore, we found that nivolumab and IL-10 differentially modulate the signal transducer and activator of transcription 3 (STAT3) and AKT serine–threonine kinase pathways. Finally, we found that nivolumab activates the mitogen-activated protein kinase (MAPK) pathway, which in turn is responsible for the observed induction of IL-10 production by nivolumab. These findings provide new insights into the mechanisms underlying anti-PD-1-modulated T cell responses by IL-10, which could lead to the discovery of novel combination treatments that target IL-10 and immune checkpoint molecules.

## 1. Introduction

Immune checkpoint inhibitors (ICIs) such as anti-programmed cell death receptor-1 (PD-1) and/or programmed cell death ligand-1 (PD-L1) therapeutic monoclonal antibodies reinvigorate the anti-tumor immunological activities by reversing immune checkpoint receptor-induced immunosuppressive effects [1,2,3,4]. The U.S. Food and Drug Administration has approved several anti-PD1 mAbs (nivolumab, pembrolizumab, cemiplimab, and dostarlimab) and anti-PD-L1 mAbs (atezolizumab, durvalumab, and avelumab) to treat cancer patients at a variety of stages due to their significant clinical benefits. However, most patients either do not respond to treatment or develop resistance to the ICI immunotherapy [5,6,7,8]. The mechanisms of unresponsiveness or resistance to this type of immunotherapy are poorly understood. Therefore, identifying factors that drive or prevent an effective T cell response to ICI immunotherapy is an urgent need for understanding these resistance mechanisms, and could lead to the discovery of novel effective combination therapies.

IL-10 has been recognized as one of the most important immunosuppressive cytokines, and accumulating evidence suggests that it has pleiotropic effects on immunoregulation and inflammation, as well as being one of the most critical modulators in anti-cancer immune responses [9,10,11]. IL-10 is widely expressed in the tumor microenvironment by tumor cells as well as various innate immune cells such as macrophages and dendritic cells. IL-10 is also highly expressed in CD4^+^ cells, CD8^+^ T cells, and regulatory T cells (Tregs) [12,13,14]. Historically, IL-10 has been thought to exert potent pro-tumor effects [15,16] mainly due to its immunosuppressive abilities [17,18]. However, recent studies also suggest that IL-10 provides significant anti-tumor effects in several mouse models. For example, tumor immune surveillance was shown to be decreased in IL-10 knockout mice, whereas transgenic overexpression of IL-10 protected mice from carcinogenesis. Furthermore, injection of PEGylated IL-10 into MMTV/HER2 transgenic mice led to tumor rejection that was dependent on activated CD8 T cells in an IFN-γ and Granzyme B-dependent manner [16].

Data from phase I/II clinical trials demonstrated that recombinant PEGylated IL-10 alone shows some anti-tumor effects against multiple cancer types including renal carcinoma, melanoma, and breast cancer [19]. In contrast, anti-IL-10 treatment increases the efficacy of an anticancer vaccine in a subset of patients [20]. Furthermore, early clinical trials demonstrated that recombinant IL-10 significantly inhibits T cell responses in both healthy donors [21] and kidney transplant patients receiving anti-CD3 mAb induction therapy [22] but does not have significant effects on autoimmune diseases such as rheumatoid arthritis and active Crohn’s disease [23,24]. Despite all of these observations, the effects of IL-10 in cancer therapy remain inconclusive due to the lack of data from large-scale clinical trials, and the scant evidence gathered from human *in vitro* testing systems.

Anecdotal evidence from animal studies suggests that IL-10 might play a role in modulating anti-PD-1/PD-L1-induced T cell responses. In a mouse ovarian cancer model, treatment with an anti-PD-1 mAb significantly increases IL-10 levels in serum and ascites. The combination of anti-IL-10 with anti-PD-1 treatment in this model significantly inhibits tumor growth compared to treatment with either component alone [25]. Consistent with these observations, blocking IL-10 increases anti-tumor T cell activity and ICI responsiveness in a chronic lymphocytic leukemia mouse model [26]. Furthermore, in a phase II clinical trial, the serum levels of IL-10 prior to treatment have been shown to be associated with better efficacy in patients treated with nivolumab [27,28]. However, these studies have not investigated whether or how IL-10 directly affects anti-PD-1-induced T cell responses. More importantly, anti-mouse PD-1 mAb treatment does not affect tumor growth when used in preclinical tumor models, indicating that these animal models do not recapitulate the heterogeneous T-cell responses of human cancers treated with anti-PD-1/PD-L1 therapy [25]. Blocking IL-10 signaling enhances anti-PD-1 induced tumor antigen-specific CD8^+^ T cell functions in metastatic melanoma patients [15]. In addition, treatment with recombinant IL-10 enhances nivolumab-induced anti-tumor activities in a small portion of these patients [29]. Nevertheless, these heterogeneous and paradoxical effects of IL-10 highlight the need to better understand the roles of IL-10 in anti-tumor responses, its impact on nivolumab-induced T cell responses, and its underlying mechanisms in eliciting these effects, especially in a human experimental system.

In the current study, using a well-established Mixed Lymphocyte Reaction (MLR) assay that has been used for the characterization of nivolumab and pembrolizumab in non-clinical studies [30,31,32], we show that treatment with either nivolumab or pembrolizumab significantly increases IL-10 production, concurrent with an increased production of the immune activation cytokine IFN-γ. Blocking IL-10 signaling induces a highly heterogeneous nivolumab-induced IFN-γ production and the expression of T cell activation markers Ki67 and CD25. Furthermore, combining nivolumab with IL-10 also impacts the expression of Granzyme B in a donor-dependent manner. Mechanistically, blocking IL-10 signaling and/or nivolumab activates various downstream signaling pathways of IL-10 and PD-1, including the STAT3 and AKT pathways. Finally, we demonstrate that nivolumab activates the MAPK pathway, leading to the increased expression of IL-10. Our study demonstrates that the induction of IL-10 by anti-PD-1 immunotherapy may be one of several possible mechanisms underlying resistance to anti-PD-1 immunotherapy.

## 2. Results

### 2.1. Treatment with Nivolumab or Pembrolizumab Induces IL-10 Cytokine Production

**Figure 1:**
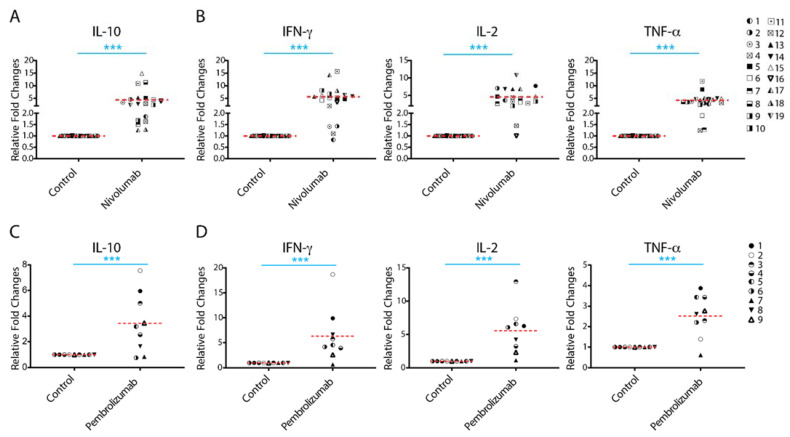
Treatment with nivolumab or pembrolizumab elicits potent interleukin-10 (IL-10) production from T cells. (**A**,**B**) Purified T cells were co-cultured with allogeneic matured monocyte-derived dendritic cells in the presence of nivolumab (20 μg/mL) for 5 days, after which the culture media was harvested for multiplex analysis of production of IL-10 (**A**(, and interferon-γ (IFN-γ), IL-2, and tumor necrosis factor-α (TNF-α) (**B**). (**C**,**D**) Purified T cells were co-cultured with allogeneic monocyte-derived dendritic cells in the presence of pembrolizumab (20 μg/mL) for 5 days, after which the culture media was harvested for multiplex analysis of the production of IL-10 (**C**) and IFN-γ, IL-2 and TNF-α (**D**). Graphs show the relative fold changes over the no-antibody treatment controls. Each symbol represents data from one individual donor pair. Note: The representative symbols between Figure 1A,B (nivolumab treatment group) and Figure 1C,D (pembrolizumab treatment group) are not the same. Student’s *t*-test, *** *p* < 0.001.

IL-10 has been recognized as one of the most potent and multifunctional immunoregulatory cytokines that has a profound effect on anti-cancer T cell responses. To analyze whether the expression of IL-10 is associated with anti-PD-1/PD-L1 immunotherapy, we performed a cytokine profiling analysis in a human allogenic MLR system using a Luminex assay detecting four cytokines: IL-10, IFN-γ, IL-2, and TNF-α. Nivolumab significantly increased production of IL-10 with 13 of 19 donor pairs showing a more than two-fold increase compared to controls (Figure 1A and Supplementary Table S1). Consistent with previous studies [33], concomitant analyses of immune activation cytokines indicated that nivolumab treatment also significantly increased the production of IFN-γ, IL-2, and TNF-α (Figure 1B and Supplementary Table S1), of which IFN-γ has been a key cytokine in IL-10 mediated anti-tumor responses [16]. Of note, while most donor pairs showed an increased level of all cytokines tested, the extent of the increase in levels of IL-10, as well as of IFN-γ, IL-2, and TNF-α varied among donor pairs.

We then analyzed the potential correlation between the increased IL-10 production and the production of other cytokines tested. We found that there is no significant correlation between the increased IL-10 production with the increased production of IFN-γ and IL-2, and only a slight correlation with the increased production of TNF-α (Supplementary Figure S1A). Interestingly, we found a significant correlation between increased IFN-γ and increased TNF-α production, whereas there is no significant correction of the increased IL-2 production with the increased IFN-γ and TNF-α production (Supplementary Figure S1B). The lack of correlation between changes in IL-2 production and production of IFN-γ and TNF-α might be due to IL-2 acting as an essential cytokine during T cell proliferation, in which IL-2 is constantly consumed by binding to IL-2 receptors on T cells.

To further confirm the effects of anti-PD-1 mAb on IL-10 production, we analyzed the effects of another anti-PD-1 therapeutic antibody, pembrolizumab, on cytokine production. Consistent with the results observed for nivolumab, pembrolizumab treatment also significantly increased production of IL-10 (Figure 1C and Supplementary Table S2), as well as the production of IFN-γ, IL-2, and TNF-α (Figure 1D and Supplementary Table S2). The levels of expression of these cytokines induced by pembrolizumab were comparable to those induced by nivolumab (Supplementary Figure S2). Similar to nivolumab treatment, the extent of the observed increase in the level of IL-10 also varied among donor pairs.

Together, our data demonstrate that both anti-PD-1 therapeutic antibodies nivolumab and pembrolizumab increase production of IL-10, as well as IFN-γ, IL-2, and TNF-α, although the extent of the cytokine responses to anti-PD-1 mAbs is significantly heterogeneous among donors.

### 2.2. Anti-IL-10 mAb Exerts Heterogeneous Effects on Nivolumab-Induced Cytokine Production

**Figure 2:**
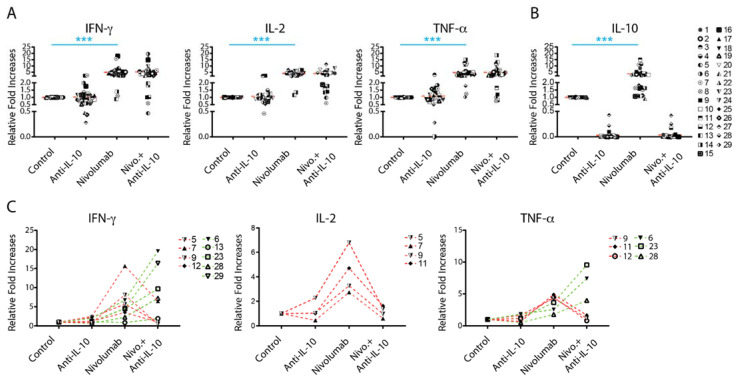
Anti-interleukin-10 (anti-IL-10) mAb induces heterogeneous cytokine responses. (**A**,**B**) Purified T cells were co-cultured with allogeneic matured monocyte-derived dendritic cells in the presence of nivolumab (20 μg/mL) with or without anti-IL-10 mAb (5 μg/mL) for 5 days, after which the culture media was harvested for multiplex analysis of the production of interferon-γ (IFN-γ), IL-2 and tumor necrosis factor-α (TNF-α), (**A**) and IL-10 (**B**). Graphics charting the relative fold change over the no-antibody treatment controls per donor pair. (**C**) Donor pairs with two-fold changes between nivolumab and nivolumab plus anti-IL10 mAb treatment are shown. Each symbol represents data from one individual donor pair. Student’s *t*-test, *** *p* < 0.001.

To explore the biological outcome of anti-PD-1 treatment-induced IL-10 production, we investigated whether blockade of IL-10 signaling affects the production of other cytokines such as IFN-γ, IL-2, and TNF-α. In comparison to controls, anti-IL-10 alone did not significantly impact the production of IFN-γ, IL-2, and TNF-α (Figure 2A). However, anti-IL-10 treatment decreased the production of IFN-γ in three donor pairs (donor pairs 21, 26, and 27) and increased production of IFN-γ in two donor pairs (donor pairs 3 and 9) by more than two-fold in comparison to controls. Similarly, anti-IL-10 treatment decreased the production of IL-2 in one donor pair (donor pair 7) and the production of TNF-α in two donor pairs (donor pairs 3 and 29) by more than two-fold, respectively. Anti-IL-10 treatment increased the production of IL-2 in two donor pairs (donor pairs 3 and 9) and the production of TNF-α in two donor pairs (donor pairs 14 and 29) by more than two-fold, respectively. Of note, anti-IL-10 mAb treatment diminished subsequent IL-10 detection, suggesting that the anti-IL-10 blocking mAb interfered with the anti-IL-10 mAb detection antibody used in the Luminex assay (Figure 2B). 

Effects of anti-IL-10 mAb on nivolumab-induced cytokine production were also heterogeneous. Whereas anti-IL-10 mAb treatment on average did not have a significant impact on nivolumab-induced cytokine production (Figure 2A), anti-IL-10 mAb treatment significantly decreased IFN-γ production in four donor pairs and decreased IL-2 and IFN-γ production in three donor pairs by more than two-fold (Figure 2C). Anti-IL-10 treatment increased the nivolumab-induced production of IFN-γ in five tested donor pairs and TNF-α in three donor pairs by more than two-fold, respectively (Figure 2C). No donor pair showed a greater than two-fold increase in nivolumab-induced IL-2 production by anti-IL-10 mAb treatment (Figure 2C).

Since the level of PD-1 expression on T cells and the status of dendritic activation may potentially affect cytokine production modulated by nivolumab, we investigated the expression of PD-1 on both CD4^+^ and CD8^+^ T cells as well as the HLA-DR expression on dendritic cells. We found that anti-IL-10 mAb significantly, but modestly, decreased expression of PD-1 on CD4^+^ and CD8^+^ T cells (Supplementary Figure S3A). However, additional anti-IL-10 mAb treatment to nivolumab did not impact the expression of PD-1 in comparison to nivolumab treatment alone (Supplementary Figure S3A). Furthermore, using HLA-DR as a dendritic activation marker [34], treatment of nivolumab and/or anti-IL-10 mAb did not impact the expression of HLA-DR on dendritic cells (Supplementary Figure S3B). Of note, the variation of expression of PD-1 and HLA-DR between different donor pairs appears modest compared to that of cytokine production. Therefore, it is unlikely that the status of expression of PD-1 and dendritic cell activation contributes to the changes in cytokine production modulated by nivolumab and/or anti-IL-10 mAb.

In summary, anti-IL-10 mAb treatment showed a heterogeneous effect on nivolumab-induced cytokine production.

### 2.3. Anti-IL-10 mAb Exerts Heterogeneous Effects on Nivolumab-Induced T Cell Activation

**Figure 3:**
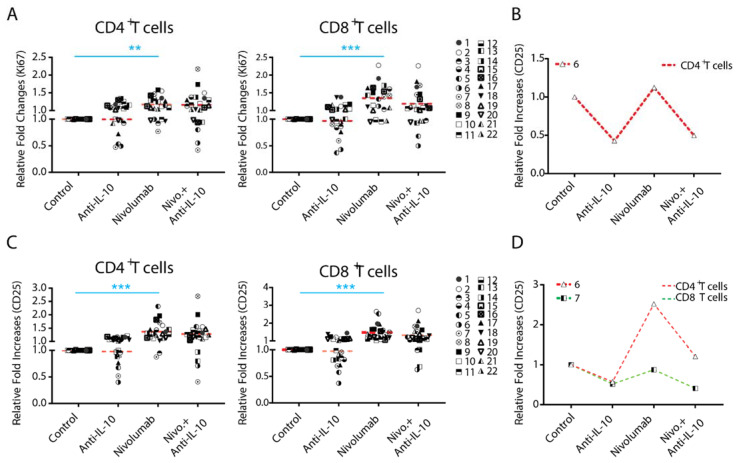
Anti-interleukin-10 (anti-IL-10) mAb induces heterogeneous expression of nuclear protein Ki67 (Ki67) and CD25 on CD4^+^ and CD8^+^ T cells. Purified T cells were co-cultured with allogeneic monocyte-derived dendritic cells in the presence of nivolumab (20 μg/mL) with or without anti-IL-10 mAb (5 μg/mL) for 5 days, after which cells were harvested for flow cytometry analysis of Ki67 expression. (**A**,**B**) Donor pairs showing that anti-IL-10 mAb decreased nivolumab-modulated expression of Ki67 in CD4^+^ and CD8^+^ T cells are shown (**A**). Donor pairs with two-fold changes between nivolumab and nivolumab plus anti-IL10 mAb treatment are shown (**B**). (**C**,**D**) Purified T cells were co-cultured with allogeneic monocyte-derived dendritic cells in the presence of nivolumab (20 μg/mL) with or without anti-IL-10 mAb (5 μg/mL) for 5 days, after which cells were harvested for flow cytometry analysis of CD25. Graphs show the relative fold change over the no-antibody treatment controls (**C**). Donor pairs showing that anti-IL-10 mAb decreased nivolumab-modulated expression of CD25 in CD4^+^ and CD8^+^ T cells are shown (**D**). Each symbol represents data from one individual donor pair. Student’s *t*-test, ** *p* < 0.01.*** *p* < 0.001.

To further define the potential role of IL-10 in T cell functions, we tested whether treatment with anti-IL-10 mAb affects the expression of T cell activation markers nuclear protein Ki67 (Ki67) and CD25. On average, treatment with anti-IL-10 mAb alone did not significantly affect expression of Ki67 and CD25 (Figure 3A,C). However, anti-IL-10 mAb treatment decreased Ki67 expression more than two-fold in two donor pairs for both CD4^+^ (donor pairs 6 and 7) and CD8^+^ (donor pairs 5 and 6) T cells (Figure 3A,C). Anti-IL-10 mAb treatment decreased expression of CD25 more than two-fold for both CD4^+^ (donor pairs 5 and 6) and CD8^+^ (donor pair 6) T cells (Figure 3A,C). No donor pairs showed an increased expression of Ki67 and CD25 for both CD4^+^ and CD8^+^ T cells by more than two-fold (Figure 3A,C).

We next analyzed the effect of anti-IL-10 mAb treatment on nivolumab-modulated Ki67 and CD25 expression. on average, anti-IL-10 mAb treatment did not impact the expression of Ki67 and CD25 induced by nivolumab. However, the effect of anti-IL-10 mAb appears heterogeneous (Figure 3A,C). Furthermore, anti-IL-10 mAb treatment decreased expression of nivolumab-modulated Ki67 in one donor pair for CD4^+^ T cells by more than two fold, but did not have an impact on CD8^+^ T cells. Anti-IL-10 mAb treatment decreased the nivolumab-modulated expression of CD25 in one donor pair for CD4^+^ T cells (donor pair 6) and one donor pair for CD8^+^ T cells (donor pair 7) by more than two-fold, respectively. 

Together, our data suggest that anti-IL-10 mAb displays an inhibitory effect on the expression of Ki67 and CD25 in a small subset of donor pairs, and does not have an additive effect in any of the donor pairs.

### 2.4. Anti-IL-10 mAb Exerts Heterogeneous Effects on Granzyme B Expression in T Cells

**Figure 4:**
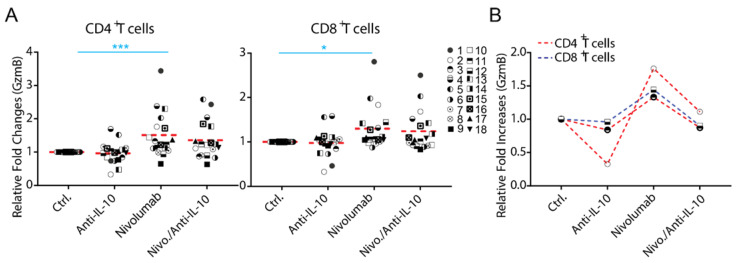
Anti-interleukin-10 (anti-IL-10) mAb induces heterogeneous expression of Granzyme B (GzmB) in CD4^+^ and CD8^+^ T cells. (**A**,**B**) Purified T cells were co-cultured with allogeneic monocyte-derived dendritic cells in the presence of nivolumab (20 μg/mL) with or without anti-IL-10 mAb (5 μg/mL) for 5 days, after which cells were harvested for flow cytometry analysis of Granzyme B expression. (**A**) Donor pairs showing that anti-IL-10 mAb decreased nivolumab-modulated expression of Granzyme B in CD4^+^ and CD8^+^ T cells are shown. Graphs show the relative fold change over the no-antibody treatment controls. (**B**) Donor pairs with two-fold changes between nivolumab and nivolumab plus anti-IL10 mAb treatment are shown. Each symbol represents data from an individual donor pair. Student’s *t*-test, * *p* < 0.05, *** *p* < 0.001.

The expression of Granzyme B (GzmB) has been shown to be a major mechanism for IL-10 mediated anti-tumor T cell responses in a mouse tumor model [16]. We examined whether blockade of IL-10 affects the expression of Granzyme B, a functional marker for T cells. We found that anti-IL-10 mAb treatment alone on average did not significantly affect the expression of Granzyme B in either CD4^+^ or CD8^+^ T cells (Figure 4A). However, anti-IL-10 mAb treatment decreased the expression of Granzyme B in two donor pairs for CD4^+^ T cells (donor pairs 2 and 13) and two donor pairs for CD8^+^ T cells (donor pairs 1 and 3) (Figure 4A).

Whereas anti-IL-10 treatment did not significantly affect the expression of Granzyme B modulated by nivolumab, the effect of anti-IL-10 mAb appears heterogeneous (Figure 4A). Furthermore, anti-IL-10 treatment decreased nivolumab-modulated expression of Granzyme B in two donor pairs for CD4^+^ T cell by more than two-fold (donor pairs 9 and 12) and did not impact the expression of Granzyme B in any of the donor pairs for CD8^+^ T cells (Figure 4B).

Together, our data suggest that anti-IL-10 mAb displays an inhibitory effect on the expression of Granzyme B, in a small subset of donors, and does not have an additive effect on nivolumab-mediated Granzyme B expression in any of them.

### 2.5. Anti-IL-10 mAb and/or Nivolumab Display Heterogeneous Effects on Activation of the AKT and STAT3 Pathways

To determine the mechanism(s) by which nivolumab-induced IL-10 production affects the nivolumab-induced T cell responses, we examined the effect of anti-IL-10 mAb and/or nivolumab on the activation of the PD-1 downstream signaling pathways such as AKT [35,36,37], as well as the IL-10 downstream signaling pathway STAT3 that has been shown to be a critical pathway modulating IL-10 mediated anti-tumor T cell responses [16].

**Figure 5:**
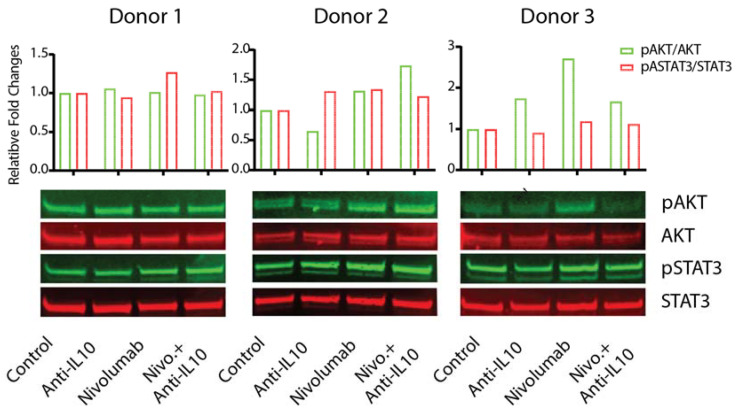
Anti-interleukin-10 (anti-IL-10) and nivolumab differentially affect activation of the AKT and signal transducer and activator of transcription 3 (STAT3) signaling pathways. Purified T cells were co-cultured with allogeneic monocyte-derived dendritic cells in the presence of nivolumab (20 μg/mL) with or without anti-IL-10 mAb (5 μg/mL) for 5 days, after which cells were harvested for WB analyses to determine activation of the AKT and STAT3 signaling pathways. Total AKT and total STAT3 were used as loading controls. Upper panel is the quantitative data of the Western blot results as conducted using ImageJ software. The activation of the AKT and STAT3 pathways is determined by the band intensity of pAKT (or pSTAT3) relative to the band intensity of total AKT (or STAT3). Data are from three donors, representative of at least eleven donor pairs.

Western Blot analyses indicated that the patterns of activation of these pathways by anti-IL-10 mAb and/or nivolumab were highly heterogeneous (Figure 5 and Supplementary Figure S4). For donor pair 1, treatments of anti-IL-10 and/or nivolumab did not impact the expression of phospho-AKT or phospho-STAT3. However, for donor pair 2, anti-IL-10 mAb treatment slightly increased the expression of phospho-STAT3 but not the expression of phospho-AKT. nivolumab, however, increased expression of both phospho-AKT and phospho-STAT3 in this donor pair. Additional anti-IL-10 mAb did not have an impact on nivolumab-modulated regulation of these pathways. Lastly, for donor 3, anti-IL-10 mAb treatment slightly decreased the expression of phospho-STAT3 but did not have an impact on the expression of phospho-AKT. Nivolumab significantly increased the expression of phospho-AKT, and slightly increased the expression of phospho-STAT3. Anti-IL-10 mAb reversed nivolumab-induced expression of phospho-AKT but did not have an impact on the expression of phospho-STAT3. In summary, our data suggest that anti-IL-10 mAb and/or nivolumab exert heterogeneous responses on activation of the AKT and STAT3 signaling pathways.

### 2.6. Nivolumab-Induced IL-10 Production Depends on the Activation of the MAPK Kinase Pathway

Many factors have been shown to regulate the expression of IL-10 in T cells, but the activation of the mitogen-activated protein kinase (MAPK) pathway has been shown to be a dominant signaling pathway that regulates the expression of IL-10 in T cells [14]. Conversely, engagement of PD-L1 with PD-1 on T cells has been shown to significantly inhibit the activation of the MAPK pathway [38,39]. 

**Figure 6:**
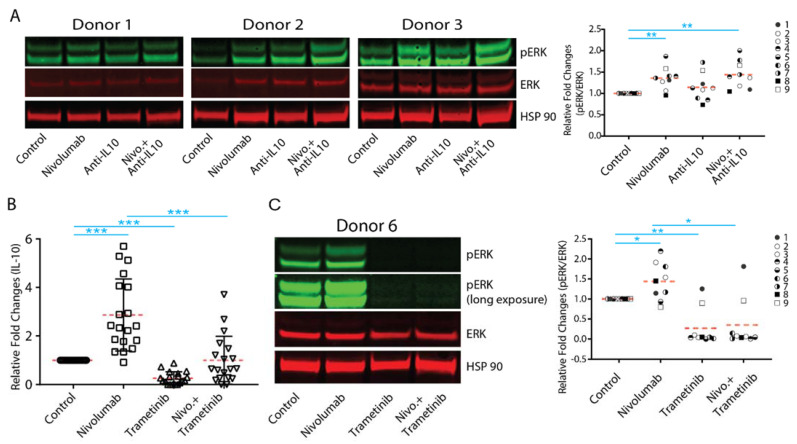
Nivolumab-mediated upregulation of interleukin-10 (IL-10) production depends on the activation of the mitogen-activated protein kinase (MAPK) pathway. (**A**,**B**,**C**) Purified T cells were co-cultured with allogeneic monocyte-derived dendritic cells in the presence of nivolumab (20 μg/mL) with or without anti-IL-10 mAb (5 μg/mL) for 5 days. (**A**) Cells were harvested for WB analyses for the MAPK signaling pathway. Shown are three donor pairs representative of 9 donor pairs. HSP90 and total ERK were used as loading controls (left panel). Quantitation of the Western blot results (combined with donor 4 to 9 is presented in Figure S5) was conducted using ImageJ software. The activation of the ERK pathway is determined by the band intensity of pERK divided by the band intensity of total ERK (right panel). Student’s t test, ** *p* < 0.01. (**B**) Purified T cells were co-cultured with allogeneic monocyte-derived dendritic cells in the presence of nivolumab (20 μg/mL) with or without trametinib (0.2 μg/mL) for 5 days. Cell culture media were harvested for Luminex analyses of IL-10 production (*n* = 19). (**C**) Purified T cells were co-cultured with allogeneic monocyte-derived dendritic cells in the presence of nivolumab (20 μg/mL) with or without trametinib (0.2 μg/mL) for 5 days. Cells were harvested for WB analyses for the MAPK signaling pathway. Shown is a Western Blot of one donor pair representative of nine donor pairs (left panel). Quantitation of the Western blot results (combined with donor 2 to 9 is presented in Figure S6) was conducted using ImageJ software. The activation of the ERK pathway is determined by the band intensity of pERK divided by the band intensity of total ERK (right panel). Student’s *t*-test, * *p* < 0.05, ** *p* < 0.01,*** *p* < 0.001.

To further elucidate the mechanistic effect of IL-10 on nivolumab-modulated T cell responses, we first examined whether nivolumab can activate the MAPK signaling pathway in T cells. We found that nivolumab significantly increased the expression of phospho-ERK, an activation marker for the MAPK pathway in T cells, in most donors (Figure 6A and Supplementary Figure S5). Interestingly, anti-IL-10 mAb treatment also increased the expression of phospho-ERK in two donor pairs (donor pair 2 and 3), and anti-IL-10 mAb treatment exerted an additive effect on nivolumab-induced expression of phospho-ERK in donor pair 2 but did not have significant effects on donor pairs 1 or 3 (Figure 6A). We then analyzed whether nivolumab-induced activation of the MAPK pathway results in the nivolumab-induced IL-10 production by trametinib, a MEK inhibitor which has been shown to effectively and specifically block activation of the MAPK pathway in T cells [40]. We found that trametinib treatment alone significantly decreased IL-10 production and inhibited nivolumab-induced IL-10 production (Figure 6B). Furthermore, trametinib treatment abolished the expression of phospho-ERK and reversed the nivolumab-upregulated expression of phospho-ERK (Figure 6C and Supplementary Figure S6). Of note, the abolished activation of the MAPK pathway by trametinib did not induce cell death (Supplementary Figure S7), indicating that trametinib-induced inhibition of cytokine production is likely due to its inhibitory effect on cell proliferation, [33] rather than cell death.

Together, our data indicate that anti-IL-10 and/or nivolumab activates the MAPK pathway in a donor-dependent manner and that nivolumab-induced IL-10 production depends on activation of the MAPK pathway.

## 3. Discussion

One of many challenges for ICI immunotherapy is the low response rate coupled with patients who develop resistance to the therapy. The mechanisms underlying these challenges are not fully understood [6,8,41,42,43,44]. IL-10 is one of the most important immunoregulatory cytokines that regulates the T cell response via modulating multiple signaling pathways [12,14,15,25,45]. In the present study, we describe that nivolumab induces potent IL-10 secretion in T cells due to activation of the MAPK pathway. The addition of anti-IL-10 mAb results in a heterogeneous T cell response and modulates many downstream signaling pathways of nivolumab and IL-10 in a donor-dependent manner. Our findings facilitate a better understanding of the mechanisms by which IL-10 modulates nivolumab-induced T cell functions which may lead to potential novel combination therapies that would improve the efficacy of, or overcome the resistance to, ICI mediated immunotherapy.

Our data show that the levels of IL-10 induced by nivolumab or pembrolizumab are highly heterogeneous. Consistent with this, our recent studies also showed that nivolumab induces IL-10 production using a PBMC-based assay [45]. This is consistent with a recent report that elevated IL-10 levels caused by anti-PD-1 mAb treatment were also found in an animal tumor model [25]. These data suggest that IL-10 may confer a heterogeneous T cell response to either nivolumab or pembrolizumab, as is observed in the clinic. Indeed, although blockade of IL-10 signaling does not significantly impact the nivolumab-modulated T cell responses, our data demonstrate that these responses are induced in a donor-dependent manner when individual donor pair data were analyzed. In terms of cytokine responses, anti-IL-10 either increased or decreased production of IFN-γ and TNF-α in a subset of donor pairs, which is also in line with the clinical data (Figure 2C). However, whereas anti-IL-10 mAb increases the level of IL-2 production, there is no evidence showing that anti-IL-10 mAb increases nivolumab-induced IL-2 production (Figure 2C). Similar to IL-2, anti-IL-10 mAb only decreases and does not increase, expression of other T cell activation markers induced by nivolumab, such as Ki67, CD25 (Figure 3B,C), and Granzyme B (Figure 4B). Interestingly, blockade of IL-10 signaling alone also exerts similar heterogeneous T cell responses. Therefore, our data reflect multiple aspects observed in clinical studies and may partially explain why some cancer patients respond to PEGylated IL-10 treatment or IL-10 in combination with anti-PD-1 immunotherapy, while others do not. However, the clinical data of PEGylated IL-10 treatment with or without anti-PD-1 immunotherapy should be interpreted cautiously due to the limited patient sample size and because this trial was designed as a single-arm trial [29]. Future studies using large-scale randomized clinical trials will validate whether PEGylated IL-10 treatment does indeed enhance the efficacy of anti-PD-1 immunotherapy. Of note, and consistent with our previous studies, the cytokine responses are more sensitive to nivolumab treatment compared to other T cell activation markers analyzed by flow cytometry [33].

The engagement of IL-10 with its receptor activates multiple signaling pathways, particularly the JAK-STAT3 pathway [12,13,46], similarly, nivolumab induces the activation of the AKT pathway, resulting in the alteration of T cell responses. Our data show that the activation of several major downstream signaling pathways by anti-IL-10 and/or nivolumab is also heterogeneous. While some donor pairs showed that anti-IL-10 mAb and/or nivolumab alters the activation of the STAT3 and AKT signaling pathways (Figure 5 and Supplementary Figure S4), other donor pairs showed different activation patterns (Figure 5 and Supplementary Figure S4) indicating that neither these two known pathways alone contribute to the heterogeneous T cell responses. These data also indicate the possibility that other pathways might play a role in the heterogeneous T responses. For example, IL-10 has been shown to regulate activation of the NF-κB pathway, [13,47] Additionally, the expression levels of IL-10 receptors on T cells between different donors might also contribute to the heterogeneous responses modulated by IL-10. Future work to establish a direct connection between the activation of IL-10 and PD-1 signaling pathways and T cell responses is warranted. 

Our study demonstrates that activation of the MAPK pathway not only regulates the nivolumab-induced signaling pathways, but it is also a consequence of activation of nivolumab downstream signaling pathways. Unlike activation of the AKT and STAT3 pathways, upregulation by nivolumab occurs in most donor pairs (Figure 6A,C and Supplementary Figures S4 and S5), and blocking of activation of the MAPK pathway significantly decreases IL-10 production (Figure 6 and Supplementary Figure S6), indicating that activation of the MAPK pathway might play a potential role in regulating the heterogeneous T cell responses caused by anti-IL-10 mAb and/or ICI immunotherapy. Collectively, our study suggests that the differential activation of multiple signaling pathways likely contributes to the heterogeneous T responses modulated by anti-IL-10 mAb and/or nivolumab treatment. Future work to identify the relevant signaling pathways modulating the effects of nivolumab and/or anti-IL-10 mAb on T cell responses will facilitate a greater understanding of the mechanism of action that could guide the identification of biomarker(s) for the clinical application of combination therapy of ICI and IL-10.

Although our study demonstrates the important roles of IL-10 and delineates the signaling pathways associated with anti-IL-10 and/or nivolumab on modulating nivolumab-induced T cell responses, there are several important aspects that need to be further investigated. (1) The serum levels of IL-10 prior to treatment have been shown to be associated with better outcomes for patients treated with nivolumab [27]. The reasons for this are likely due to the immunostimulatory effects of IL-10 that prevail over its immunosuppressive functions in these patients with high endogenous levels of IL-10. Nonetheless, additional IL-10 treatment might not be beneficial for these patients, and in some cases, might even potentially suppress T cell responses as demonstrated in our functional assessment in which many donor pairs show increased cytokine production by anti-IL-10 treatment in the absence or presence of nivolumab (Figure 2B,D). Therefore, it is critical to identify predictive biomarkers in patients that respond to IL-10 monotherapy alone, as well as in combination with nivolumab [15,48]. (2) The experimental system we used in this study focuses on CD4^+^ and CD8^+^ T cell functions. IL-10 not only plays an important role in modulating T cell functions but also plays critical roles in modulating anti-tumor or pro-tumor effects by other immune cells that are abundant in the tumor microenvironment. These immune cells include different types of helper T cells, other types of immune cells such as macrophages, NK cells, NKT cells, myeloid-derived suppressor cells (MDSC), etc., all of which have been shown to play critical roles in anti-cancer immune responses [6,49,50,51,52]. The development of an experimental system that contains these immune cells, as well as tumor cells, to better reflect the true complexity of the tumor microenvironment will be key to further delineating the mechanism of IL-10 in regulating ICI immunotherapy. Our ongoing studies of the roles of IL-10 and/or nivolumab in regulating the function of other types of helper T cells such as regulatory T cells and other types of cells such as NK cells among others are an effort to shed more light on the roles and mechanisms of IL-10 in anti-cancer immune responses. (3) Although we demonstrate that the involvement of several signaling pathways pertains to the roles of IL-10 in nivolumab-mediated T cell responses, we did not identify the specific pathway or pathways that determine the fate of heterogeneous T cell responses modulated by anti-IL-10 and/or nivolumab. Since the T cell responses modulated by anti-IL-10 and/or nivolumab are highly heterogeneous and may spatially and temporally be controlled through convergence or divergence of multiple downstream signaling pathways, future work is needed to identify the key critical signaling pathways by unbiased approaches such as next-generation sequencing (NGS) or proteomics in addition to exploring other signaling pathways modulated by PD-1 such as the SHP2 and TCR signaling pathways [35,36,53]. These studies will not only reveal additional mechanisms critically important for future clinical application, but they will also facilitate finding novel combination therapies that are more specific to targeting IL-10 or its downstream pathways, and thus able to exclusively enhance the immunostimulatory anti-tumor effects while excluding the immunosuppressive functions of IL-10.

In conclusion, our study not only illustrates how nivolumab-induced IL-10 production may shape the T cell responses via modulating multiple downstream signaling pathways, but also provides a rationale for the combination of targeting IL-10 and PD-1 for cancer patients. Future work on dissecting the mechanisms using more comprehensive approaches on how IL-10 and/or nivolumab modulate anti-cancer immune responses in the context of the tumor microenvironment, will lead to identifying biomarkers and discovering more effective anti-cancer immunotherapies targeting IL-10 and PD-1.

## 4. Materials and Methods

### 4.1. PBMCs and Monocyte Derived Dendritic Cells

This study—using human peripheral blood mononuclear cells (PBMCs) and monocytes—was reviewed and approved by both the National Institutes of Health and the Food and Drug Administration Internal Review Boards. The demographic information of donors is listed in Supplementary Table S1. Human peripheral blood mononuclear cells (PBMCs) were obtained from healthy human volunteers by leukophoresis and purified from whole blood by ficoll-hypaque sedimentation. Monocytes were purified from PBMCs by countercurrent centrifugal elutriation [54]. PBMCs and monocytes were frozen at concentrations of 50 × 10^6^ and 20 × 10^6^, respectively, in freezing medium containing 90% fetal bovine serum (Cat#BS3032, Valley Biomedical, Winchester, VA, USA) and 10% DMSO (Cat#D8418, Sigma-Aldrich, St. Louis, MO, USA). T cells were enriched from PBMCs using the RoboSep cell isolation platform (#19051, StemCell Technologies, Vancouver, Canada). Isolated monocytes were differentiated into dendritic cells by culturing at a concentration of 1 × 10^6^ cells per mL in complete RPMI1640 (Cat#11875-093, Thermo Fisher Scientific, Waltham, MA, USA) containing 10% human AB serum (Cat#HP1022, Valley Biomedical), 1% Penn/Strep (#15140, Thermo Fisher Scientific), 1% HEPES (Cat#15630-080, Thermo Fisher Scientific), 0.1 mM 2-Mercaptoehanol (Cat#M6250, Sigma-Aldrich), 50 ng/mL GM-CSF (Cat#215-GM/CF, R&D Systems, Minneapolis, MN, USA), and 20 ng/mL IL-4 (Cat#204-IL/CF, R&D Systems) for 7 days as previous described [33]. On day 6 of culture, dendritic cells were matured with 100 ng/mL LPS (Cat#L2880, Sigma-Aldrich) for 24 h. All donor pairs used in this manuscript are listed in Table S1.

### 4.2. Mixed Lymphocyte Reaction

For the mixed lymphocyte reaction (MLR), monocyte-derived dendritic cells were harvested and resuspended at a concentration of 2 × 10^5^ cells/mL in complete RPMI medium (Cat#11875-093, Invitrogen) containing 5% human AB serum, 1% Penn/Strep, 1% HEPES, and 0.1 mM 2-Mercaptoethanol at 1 × 10^5^ cells/mL. Allogeneic T-cells were co-cultured with the matured dendritic cells at a concentration of 1 × 10^6^ cells/mL in 96 well plates (Cat#3595, Corning, NY 14831 USA) for 5 days in the presence or absence of nivolumab and pembrolizumab (20 μg/mL, via McKesson Specialty Health, Scottsdale, Arizona), and functional anti-IL-10 monoclonal neutralizing antibody (Cat#AHC0103, clone JES3-9D7, Thermo Fisher Scientific) (5 μg/mL). After 5 days, cell culture supernatants were harvested for Luminex cytokine analysis, and cells were harvested for flow cytometry analysis as well as for Western blot protein analysis. 

### 4.3. Cytokine Luminex Assay

Cytokine assays on cell culture supernatants were performed in duplicate using multiplex bead-based kits (eBioscience, San Diego, CA, USA) for the indicated cytokines as per the manufacturer’s instructions. The fluorescence of beads was measured using a BioPlex 200 analyzer (Bio-Rad Laboratories, Hercules, CA, USA). Cytokine data analysis was performed using the BioPlex Manager software (v. 6.2, BioHercules, CA). Concentrations were determined using a 5-parametric logistic nonlinear regression curve-fitting algorithm. 

### 4.4. Flow Cytometry

Cells were harvested and stained with Live/Dead Aqua (Cat#L34966, Invitrogen, Eugene, OR, USA), prepared according to the manufacturer’s instructions. Once cells were washed in PBS containing 5% fetal bovine serum and 0.1% sodium azide (Cat#26628-22-8, Sigma-Aldrich, St. Louis, MO, USA), they were fixed and permeabilized using eBioscience FoxP3 Fixation/Permeabilization kit (Cat#00-5521-00, Thermo Fisher Scientific). Cells were then stained for both extracellular and intracellular antigens using fluorescence-conjugated antibodies to human CD3 (BV711 Clone UCHT1, Cat#563725, BD Biosciences, San Jose, CA 95131, USA), CD4 (FITC Clone RPA-T4, Cat#561842, BD Biosciences), CD8 (BV650 Clone RPA-T8, Cat#563821, BD Biosciences), CD25 (BV605 Clone 2A3, Cat#562660, BD Biosciences), Granzyme B (BV421 Clone GB11, Cat#563389, BD Biosciences), PD1 (BV421 Clone MIH4, Cat#562323, BD Biosciences), Ki67 (Alexa Fluor 700 Clone B56, Cat#561277, BD Biosciences), and HLA DR (AF700 Clone G46-6 Cat# 560743 BD Biosciences). Flow cytometry analysis was performed using a 5-laser BD LSR Fortessa™ flow cytometry system, and data were analyzed with FlowJo software (v. 10.7.1, BD Biosciences). For analysis of expression of HLA-DR on dendritic cells, CD3-negative cells were gated for dendritic cells.

### 4.5. Western Blot Analysis

T cells were gently harvested from the dendritic and T cell co-cultures that were treated with nivolumab and/or anti-IL-10 mAb for Figure 5, or MEK inhibitor trametinib (GSK1120212, 0.2 μM, Cat# HY-10999, Selleckchem, Houston, TX, USA) and/or nivolumab for Figure 6 for five days. Cells were washed twice with cold PBS and cell pellets were lysed using NuPAGE LDS sample buffer (Cat#NP0008, Invitrogen). Lysates were separated on 4–12% Tris-glycine gels (Invitrogen) and were transferred to nitrocellulose membranes. Anti phospho-STAT3 (Cat#9145S), -STAT3 (Cat#9139S), -phospho-AKT (Cat#4060L), -AKT (Cat#9272S), -phospho-ERK (Cat# 9107) and -ERK (Cat#9107) Abs were used as primary antibodies (Cell Signaling Technology). HSP90 (Cat#4877S) was used as a loading control (Cell Signaling Technology). Membranes were blocked with Odyssey Blocking Buffer (Cat#927-60001, LI-COR Biosciences, Lincoln, NE, USA) and incubated overnight at 4 °C with the respective antibody as per the manufacturer’s recommendations. Donkey anti-mouse (680nm Cat#926-68072, LI-COR Biosciences) or donkey anti-rabbit IRDye (800nm Cat#926-32213, 680nm Cat#926-68023, LI-COR Biosciences at 1:10,000) were used as secondary antibodies and incubated for one hour at room temperature. Following washing, the blots were scanned and analyzed using the Odyssey Classic Imaging Detection System (LI-COR Biosciences). Quantification of WB data was performed with ImageJ software (v. 1.83e). The bands of interest were manually circled. The area and average intensity of the circled bands was calculated by the software. Similar circles in the adjacent area without bands were selected as controls. The intensity of bands was calculated by subtracting the control from the intensity of the phospho- or total targeted protein [55].

### 4.6. Statistical Analysis

Statistical analysis was performed using GraphPad Prism software (v. 9, GraphPad Software, San Diego, CA, USA). A two-tailed Student’s *t-*test was used to analyze differences in cytokine production and expression of T cell activation markers among the experimental groups. Regression analysis was performed to determine the correlation between cytokine production after treatment. A *p*-value of <0.05 was considered to be statistically significant. 

## Data Availability

The datasets used and/or analyzed for this study will be made available upon reasonable request to the corresponding authors.

## Supplementary Materials

Supplementary materials can be found at www.mdpi.com/article/10.3390/ijms222111848/s1.
