# Supplementary material for: IL-10 Signaling Elicited by Nivolumab-Induced Activation of the MAP Kinase Pathway Does Not Fully Contribute to Nivolumab-Modulated Heterogeneous T Cell Responses
Source: Int J Mol Sci. 2021 Oct 31;22(21):11848. doi: 10.3390/ijms222111848 (PMC8584131; doi:10.3390/ijms222111848)
Supplement: Supplementary file 1 [file ijms-22-11848-s001.zip › ijms-1370868-supplementary.pdf]

Supplementary Information

IL-10 Signaling Elicited by Nivolumab-Induced Activation of the MAKP Kinase Pathway Does Not Fully Contribute to Nivolumab-Modulated Heterogeneous T Cell Responses

Taylor A. Harper, Silvia M. Bacot, Christie Jane Fennell, Rebecca L. Matthews, Christina Zhu, Peng Yue, Alexander Benton, Devira Friedman, Adovi Akue, Mark A. KuKuruga, Shiowjen Lee, Tao Wang, Gerald M. Feldman

Supplementary Table S1. Demographic information for donor pairs presented in Figure S1A.

Donor pairs	DC Donor ID	Age	Sex	Race	T cell Donor ID	Age	Sex	Race
1	W092115050831	41	Male	African American	W092116050933	51	Male	Caucasian
2	W092115050831	41	Male	African American	W092116051083	58	Female	African American
3	W092115050831	41	Male	African American	W092116051093	69	Male	African American
4	W092115050703	23	Male	African American	W092116051093	69	Male	African American
5	W092116050702	48	Male	Caucasian	W092115051435	32	Male	Caucasian
6	W092116050702	48	Male	Caucasian	N/A			
7	W092118051090	28	Male	Caucasian	W092116050915	29	Male	African American
8	W092118051090	28	Male	Caucasian	W092115051506	23	Female	Caucasian
9	W092116050702	48	Male	Caucasian	W092117050506	67	Male	Caucasian
10	W092116050702	48	Male	Caucasian	W092116050915	29	Male	African American
11	W092116050702	48	Male	Caucasian	W092116050933	51	Male	Caucasian
12	W092116050702	48	Male	Caucasian	W092116050884	24	Female	Caucasian
13	W092118051076	36	Female	Caucasian	W092115051016	43	Male	Spanish
14	W092118051076	36	Female	Caucasian	W092115051265	64	Male	Caucasian
15	W092118051076	36	Female	Caucasian	W092115051355	40	Male	Caucasian
16	W092118051076	36	Female	Caucasian	W092115051596	47	Male	African American
17	W092118050945	29	Female	Caucasian	W092116050884	24	Female	Caucasian
18	W092118050945	29	Female	Caucasian	W092118051063	59	Male	African American
19	W092118050945	29	Female	Caucasian	W092116051093	69	Male	African American

Note: N/A not available.

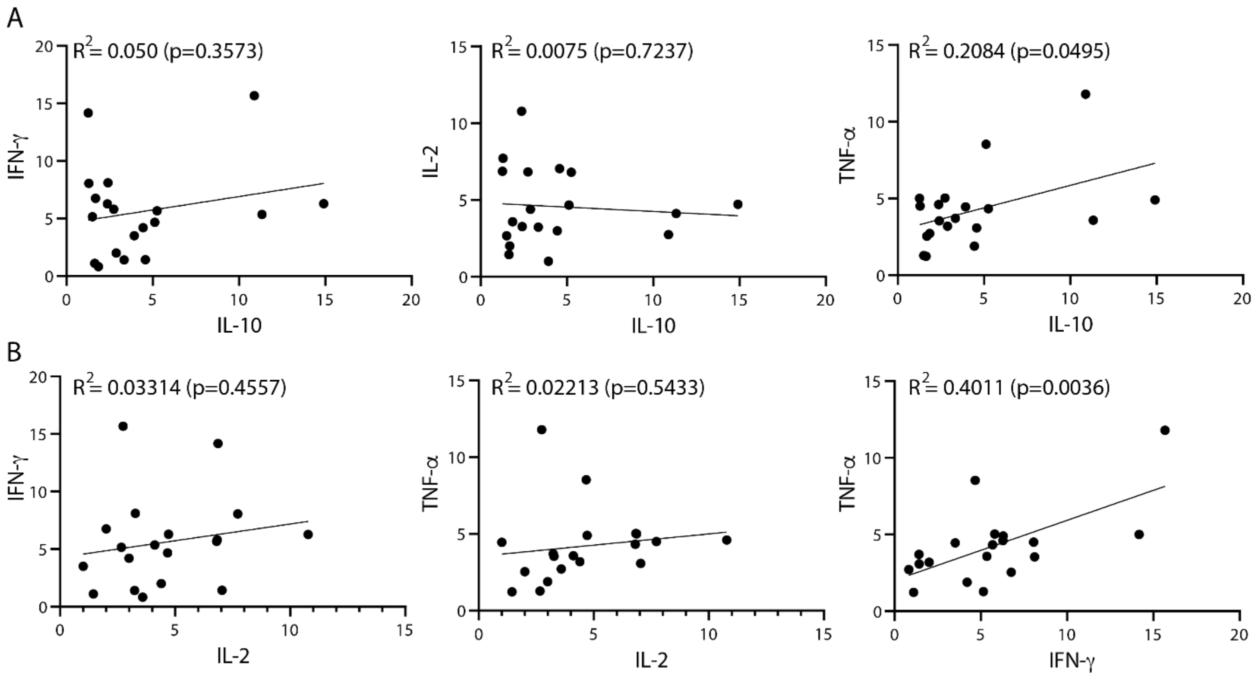

Supplementary Figure S1. Correlation of cytokine production after nivolumab treatment. (A, B). Purified T cells were co-cultured with allogeneic matured monocyte-derived dendritic cells in the presence of nivolumab (20 μg/mL) for 5 days, after which the culture media was harvested for multiplex analysis of productions of IL-10 and IFN-γ, IL-2 and TNF-α. Simple linear regression analysis was performed to determine the correlation between IL-10 and the production of IFN-γ, IL-2 and TNF-α **(A),** as well as the correlation between IFN-γ, IL-2 and TNF-α production. **(B)**.

Each symbol represents data from one individual donor pair.

Supplementary Table S2. Nivolumab increases production of cytokines (Raw data for Figure S1A)

Cytokines / Donor pair	IL-10 (pg/mL)		IFN-γ (pg/mL)		IL-2 (pg/mL)		TNF-α (pg/mL)	
	Control	nivolumab	Control	nivolumab	Control	nivolumab	Control	nivolumab
Pair 1	51.83	96.03	5206.07	4322.2	143.56	514.49	60.24	164.04
Pair 2	53.72	245.44	4430.7	6325.69	39.21	276.01	132.93	408.83
Pair 3	10.76	35.97	1475.79	2086.78	42.28	136.79	23.02	85.21
Pair 4	15.02	43.29	3811.12	7638.13	24.15	106.24	24.92	79.46
Pair 5	24.13	36.37	1124.36	5251.23	112.436	525.123	38.7	330.39
Pair 6	6.53	73.95	1085.44	4564.67	154.35	462.37	86.28	163.6
Pair 7	9.765	23.54	167.79	1361.09	179.12	477.19	133.55	170.81
Pair 8	3.79	6.4	184.885	1249.08	146.76	603.61	79.23	284.18
Pair 9	23.115	251.64	334.02	5235.71	16.925	55.37	10.93	38.79
Pair 10	26.235	42.91	1412.22	1560.55	10.1	20.25	18.285	46.41
Pair 11	10.42	54.73	1409.99	8009.13	16.555	45.36	12.355	145.78
Pair 12	29.17	79.77	1608.46	9333.87	3.78	5.48	62.955	77.83
Pair 13	16.07	239.56	2571.94	16182.95	3.33	22.66	35.88	155.6
Pair 14	6.83	26.84	1290.18	4535.45	2.84	19.39	64.7	325.33
Pair 15	43.69	54.93	380.87	5396.9	1.74	8.2	59.26	290.97
Pair 16	43.93	56.73	726.22	5855.84	2.84	2.84	29.94	133.53
Pair 17	77.07	182.42	1230.54	7729.6	5.37	36.86	48.7	243.89
Pair 18	51.83	96.03	5206.07	4322.2	14.74	113.78	51.23	231.06
Pair 19	53.72	245.44	4430.7	6325.69	6.58	70.95	50.64	233.22

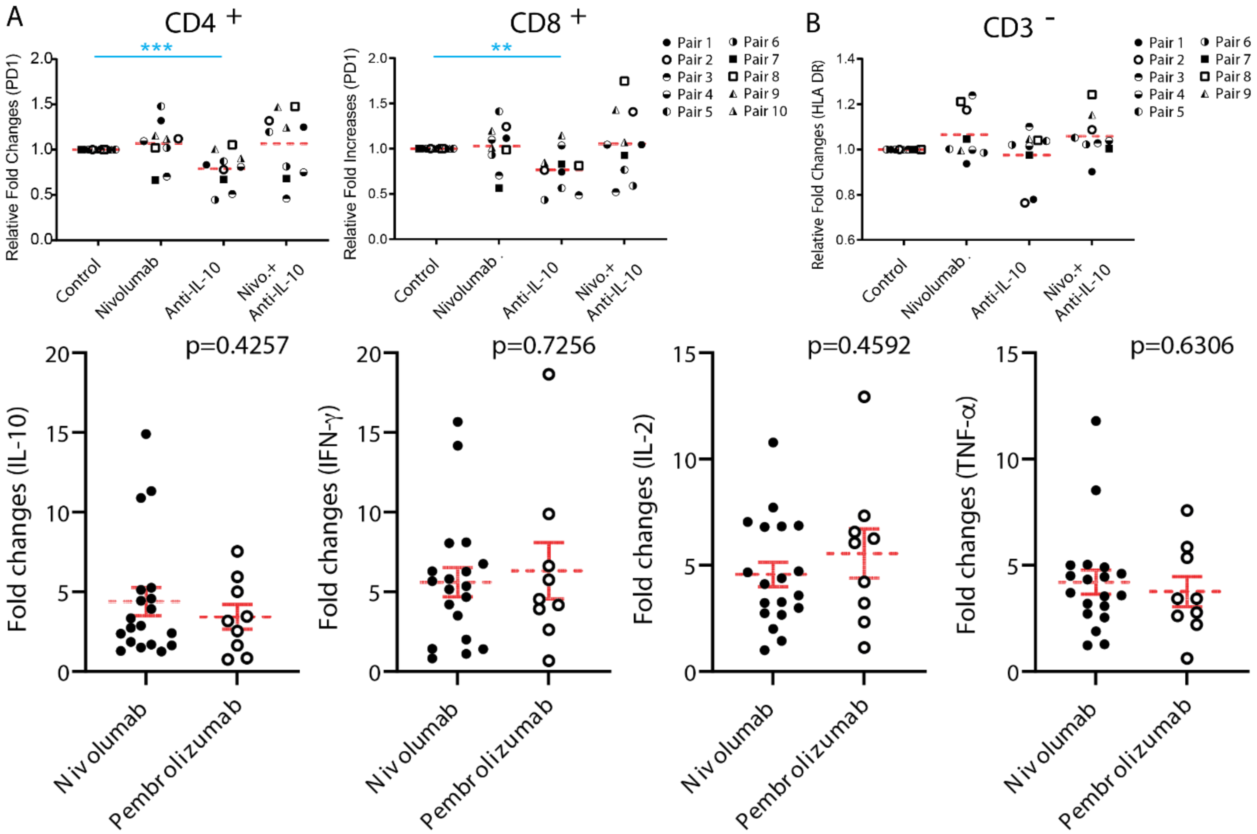

Supplementary Figure S2. Nivolumab and pembrolizumab induce comparable levels of cytokine production. Purified T cells were co-cultured with allogeneic matured monocyte-derived dendritic cells in the presence of nivolumab (20 μg/mL) and pembrolizumab (20 μg/mL) for 5 days, after which the culture media was harvested for multiplex analysis of production of IL-10, IFN-γ, IL-2 and TNF-α.

Supplementary Table S2B. Pembrolizumab increases production of cytokines (Raw data for Figure S1)

Cytokines / Donor Pair	IL-10 (pg/mL)		IFN-γ (pg/mL)		IL-2 (pg/mL)		TNF-α (pg/mL)	
	Ctrl.	pemb.	Ctrl.	pemb.	Ctrl.	Pemb.	Ctrl.	pemb.
pair 1	72.14	183.39	1695.81	6647.79	*1.78	5.73	109.95	377.35
pair 2	16.77	53.38	537.2	2441.92	*2.54	16.69	45.54	156.37
pair 3	45.12	34.07	825.9	3448.74	*2.29	13.86	80.73	178.08
pair 4	20.56	17.33	1066.15	712.01	5.19	5.9	63.84	39.74
pair 5	34.52	56.81	726.51	4812.22	*2.29	9.67	52.72	137.95
pair 6	20.06	69.43	1907.28	4999.89	*1.19	*2.77	28.99	80.45
pair 7	30.11	179.07	1277.15	12643.66	OOR <	*3.09	86.94	660.06
pair 8	11.42	86.21	873.92	16310.37	*1.41	11.19	145.83	776.48
pair 9	26.19	131.03	2151.09	12402.68	7.04	91.01	126.63	738.22

OOR< = Out of (lower) Range

*Value = Value extrapolated beyond standard range

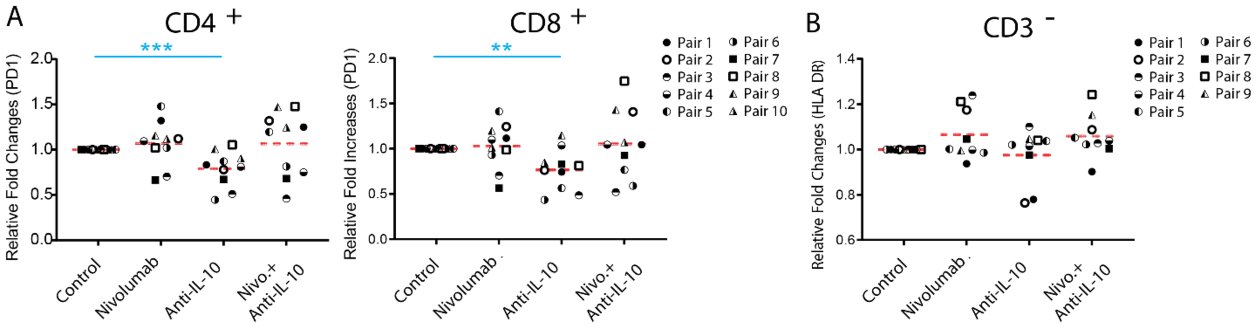

Supplementary Figure S3. Effect of nivolumab and anti-IL-10 on expression of PD-1 on CD4⁺ and CD8⁺ T cells, as well as HLA-DR on dendritic cells during MLR. (A&B) Purified T cells were co-cultured with allogeneic matured monocyte-derived dendritic cells in the presence of nivolumab (20 µg/mL) and/or anti-IL-10 mAb (5 µg/mL) for 5 days. **(A)** Changes in expression of PD-1 on $CD4^+$ T cells (left panel) and $CD8^+$ T cells (right panel) were measured by flow cytometry. **(B)** Changes in expression of HLA-DR on dendritic cells (gated on CD3 negative cells) were measured by flow cytometry.

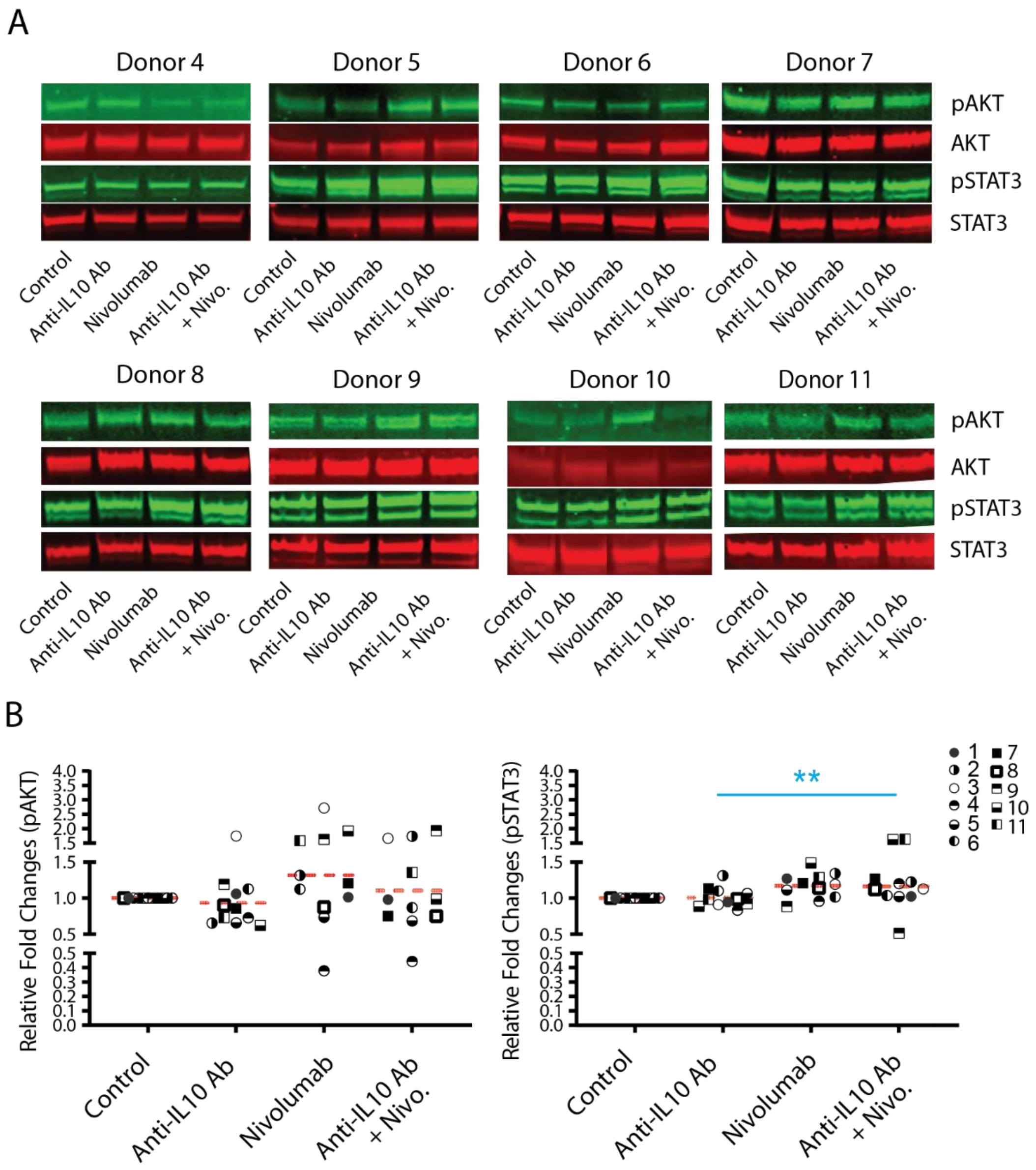

Supplementary Figure S4. Anti-IL-10 mAb and nivolumab differentially affects activation of the STAT3 and AKT signaling pathways. (A) Purified T cells were co-cultured with allogeneic monocyte-derived dendritic cells in the presence of nivolumab (20 μg/mL) with or without anti-IL-10 mAb (5 μg/mL) for 5 days, after which cells were harvested for Western blot analyses to determine activation of the AKT and STAT3 signaling pathways. Total AKT and total STAT3 were used as loading controls. **(B)** Quantitation of the Western blot results (donors 1, 2 and 3 are presented in Figure 5) was conducted using ImageJ software. The activation of the AKT and STAT3 pathways is determined by the band intensity of pERK divided by the band intensity of total ERK. Each symbol represents data from one individual donor pair. Student's t test, ** $P < 0.01$.

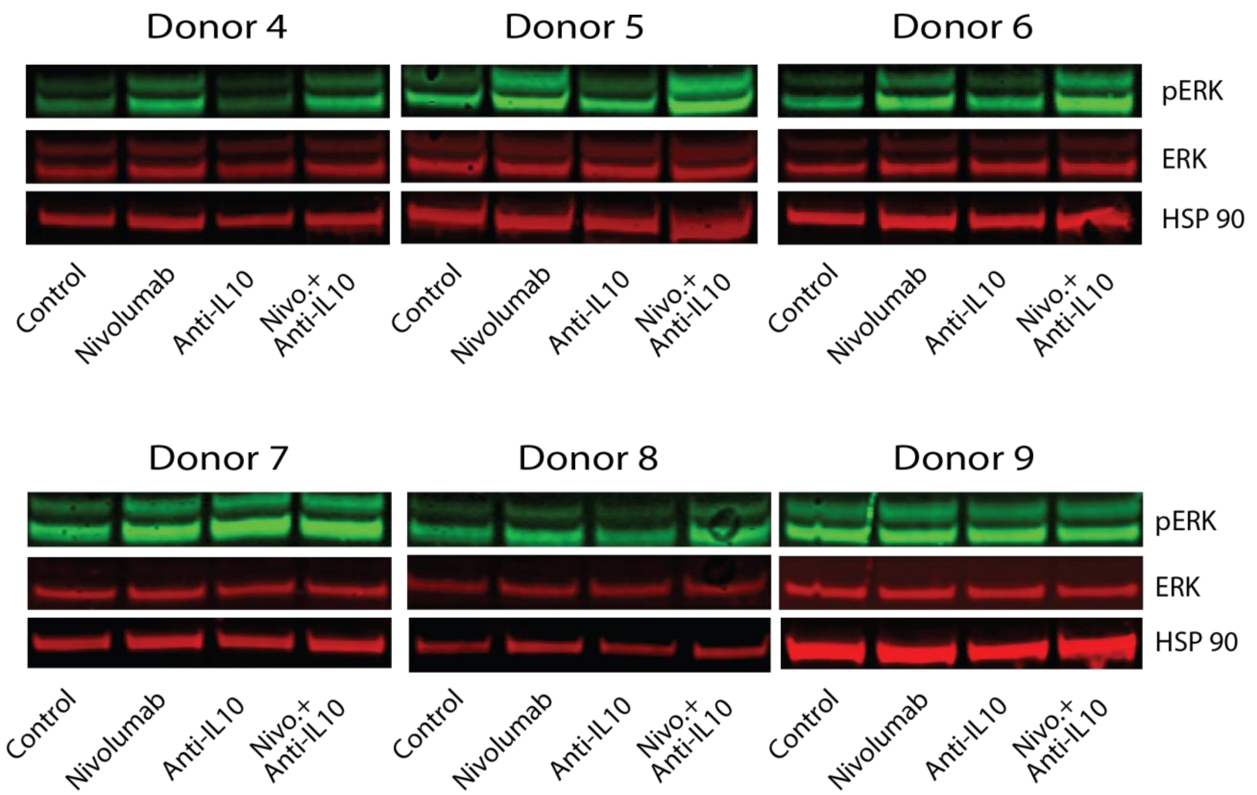

Supplementary Figure S5. Nivolumab induces activation of the MAPK pathway in T cells. Purified T cells were co-cultured with allogeneic matured monocyte-derived dendritic cells in the presence of nivolumab (20 μg/mL) and/or anti-IL-10 mAb (5 μg/mL) for 5 days. Cells were harvested for Western blot analyses to determine activation of the MAPK signaling pathway.

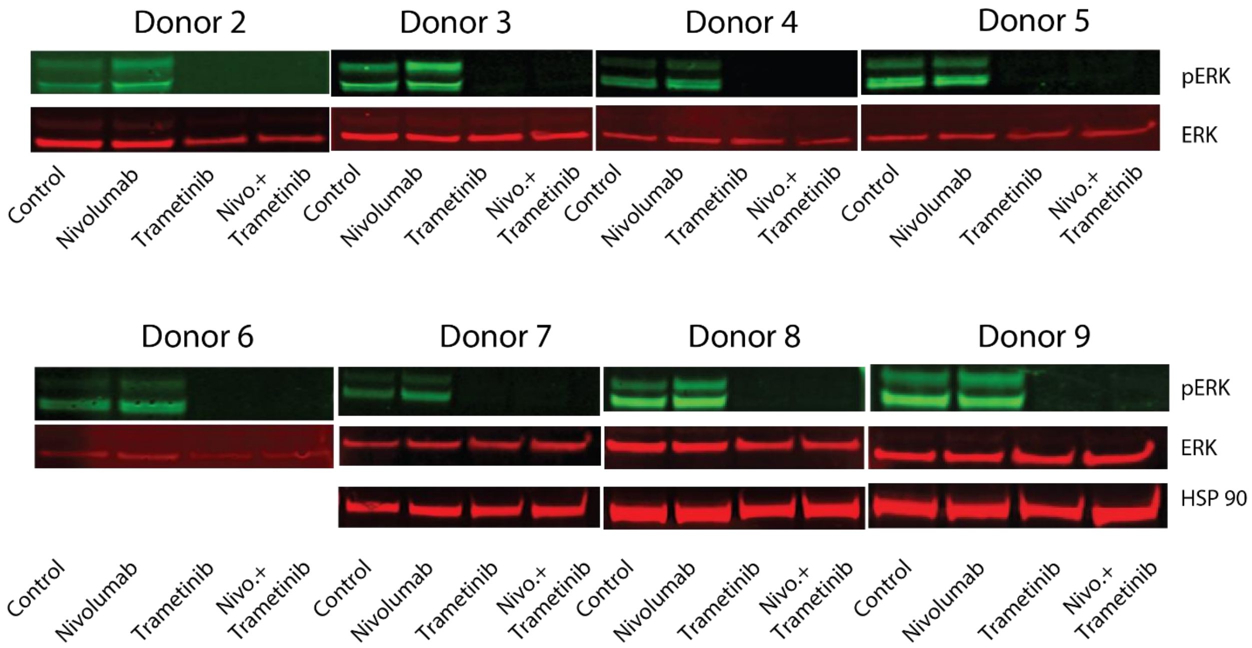

Supplementary Figure S6. MEK inhibitor diminishes nivolumab-induced activation of the MAPK pathway. Purified T cells were co-cultured with allogeneic monocyte-derived dendritic cells in the presence of nivolumab (20 µg/ml) with or without trametinib (0.2 µg/mL) for 5 days. Cells were harvested for Western blot analyses to determine activation the MAPK signaling pathway.

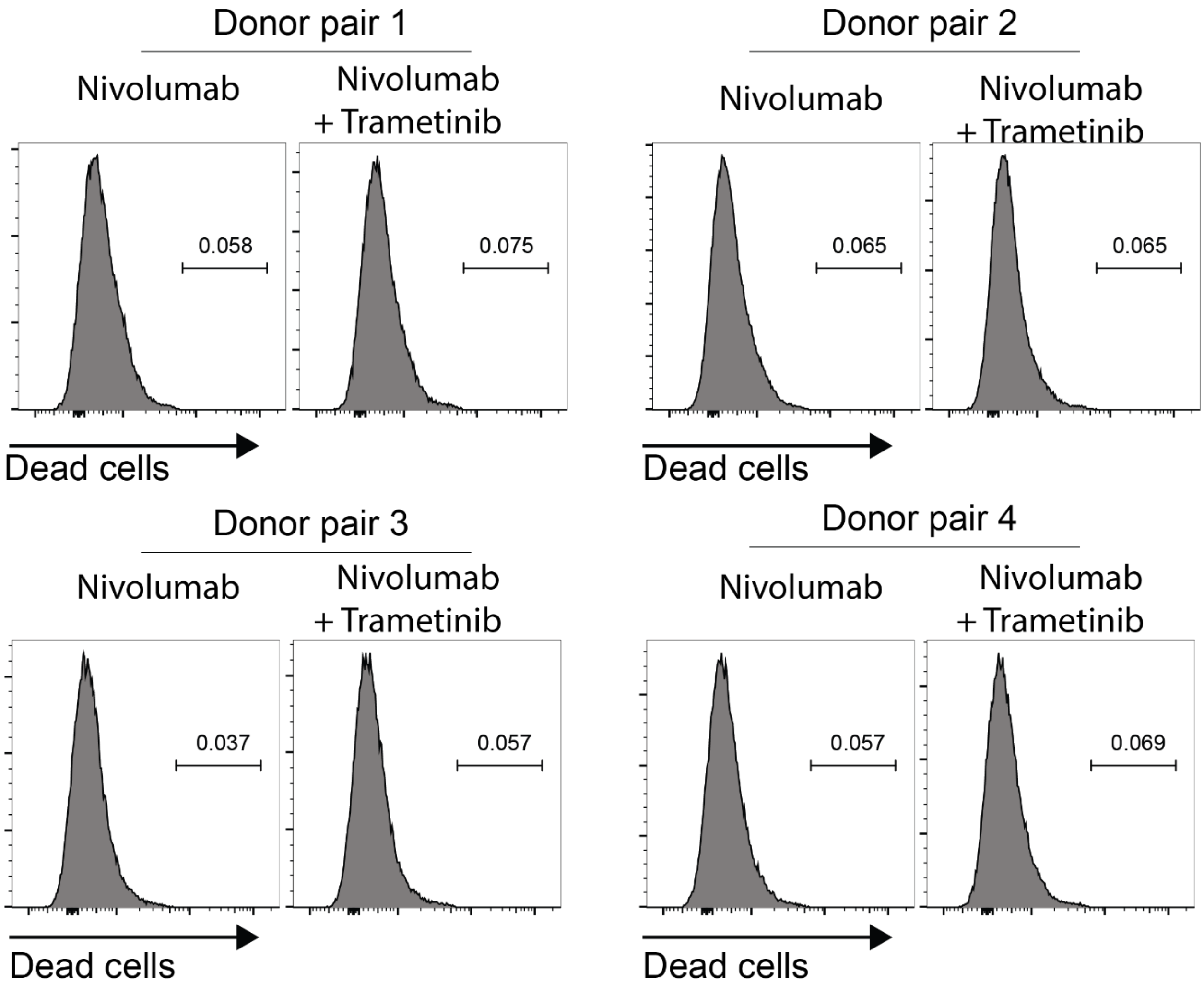

Supplementary Figure S7. MEK inhibitor does not induce cell death in T cells. Purified T cells were co-cultured with allogeneic monocyte-derived dendritic cells in the presence of nivolumab (20 μg/mL) with or without trametinib (0.2 μg/mL) for 5 days. Cells were harvested for flow cytometry analyses to determine cell death. Live/Dead Aqua was used as a viability marker.
